# *Akkermansia muciniphila* reduces susceptibility to *Listeria monocytogenes* infection in mice fed a high-fat diet

**DOI:** 10.1080/19490976.2023.2229948

**Published:** 2023-07-10

**Authors:** Jonathan M. Keane, Vanessa Las Heras, Jorge Pinheiro, Jamie A. FitzGerald, María A. Núñez-Sánchez, Cara M. Hueston, Liam O’Mahony, Paul D. Cotter, Colin Hill, Silvia Melgar, Cormac G. M. Gahan

**Affiliations:** a APC Microbiome Ireland, University College Cork, Cork, Ireland; b School of Microbiology, University College Cork, Cork, Ireland; c Teagasc Food Research Centre, Moorepark, Ireland; d Obesity and Metabolism Laboratory, Biomedical Research Institute of Murcia (IMIB-Arrixaca), Murcia, Spain; e Department of Medicine, University College Cork, Cork, Ireland; f School of Pharmacy, University College Cork, Cork, Ireland

**Keywords:** *Akkermansia muciniphila*, *Listeria*, high-fat diet, infection, resistance, inflammation, goblet cells, microbiome, metabolites

## Abstract

A high-fat (HF) diet reduces resistance to the foodborne pathogen *Listeria monocytogenes*. We demonstrate that short-term gavage with *A. muciniphila* increases resistance to oral and systemic *L. monocytogenes* infection in mice fed a HF diet. *A. muciniphila* reduced inflammation in the gut and liver of mice fed a high-fat diet prior to infection and reduced inflammatory cell infiltration in the ileum to levels similar to mice fed a low-fat (LF) diet. *Akkermansia* administration had minimal impacts upon the microbiota and microbial metabolites and did not affect individual taxa or impact the Bacteroidetes to Firmicutes ratio. In summary, *A. muciniphila* increased resistance to *L. monocytogenes* infection in mice fed a HF diet by moderating immune/physiological effects through specific interaction between *A. muciniphila* and the host gut.

## Introduction

A Westernized diet in which a high proportion of caloric intake is derived from animal fats is known to influence microbiota community structure and immune-inflammatory responses in the host.^1,2^ As well as being associated with the development of obesity, type 2 diabetes and inflammatory bowel disease, this diet can also increase susceptibility to foodborne pathogens including *Salmonella* Typhimurium and *Listeria monocytogenes* in animal models.^1,3,4^ We previously demonstrated that a high-fat (HF) diet induces inflammatory gene expression in the gut prior to *Listeria* exposure and increases susceptibility to oral and systemic *Listeria* infection.^3^ This may be due to an increased number of goblet cells in the colonic mucosa of these mice which are known to facilitate interaction between listerial Internalin A and host E-cadherin and MET. Other broader effects on immune/inflammatory responses were also evident.^5^ HF diet also affected the gut microbiota in this model, causing a predictable increase in levels of Firmicutes (including *Coprococcus*, *Butyricicoccus*, *Turicibacter* and *Clostridium XIVa)* and a reduction in Verrucomicrobiota, with these alterations to the gut microbiota subsequently exaggerated by infection with *Listeria*.^3^

The genus *Akkermansia* is a member of the phylum Verrucomicrobiota, whose type species is *A. muciniphila*.^6^ This aero-tolerant, oval-shaped, Gram-negative bacterium makes up 1–4% of the human microbiome.^7^ It uses mucin as sole source of carbon and nitrogen which allows *A. muciniphila* to thrive in nutrient-depleted environments.^6,8^ Metagenomic comparisons suggest that at least eight *Akkermansia* species can be found in the human microbiome, and concurrent colonization with different strains can occur.^9^ *A. muciniphila* localizes to the mucus layer especially in the cecum, closely associates with intestinal cells,^10^ and is predicted to produce 61 mucin-degrading proteins.^11^ It produces the short chain fatty acids acetic acid and propionate^6^ and facilitates the production of butyrate by other bacteria,^12^ which can have positive impacts on gut health.^13^ *A. muciniphila* has been shown to positively modulate host immune responses^14^ and gut barrier function^10^ and a reduced abundance later in life is associated with increased inflammation and reduced barrier function which can be rectified by butyrate supplementation.^15,16^ *A. muciniphila* is consistently reduced in humans or mice consuming a high-fat/westernized diet.^17–19^ It has been associated with a lean phenotype and reduced body weight gain.^14^ It inversely correlated with diseases such as ulcerative colitis and Crohn’s disease and has been suggested as an indicator of a healthy intestine.^20^ It may also ameliorate the metabolic disorders associated with consumption of HF diets and obesity.^17^

Based on these findings, we have investigated the effect of *A. muciniphila* on *L. monocytogenes* infection in the context of HF diet. Here we demonstrate that oral gavage with *A. muciniphila* enhances resistance to *L. monocytogenes* in mice fed a HF diet. Measured at the timepoint prior to *Listeria* infection, exposure to *A. muciniphila* ameliorated inflammatory gene expression in the distal ileum and this was coincident with a reduction in inflammatory cell infiltration. Microbiome analysis identified changes that were primarily driven by the diet, while global microbial metabolomics data failed to identify changes induced specifically by *A. muciniphila*. To our knowledge this is the first report of the effects of *A. muciniphila* against a foodborne pathogen and the results have implications for the use of microbial interventions in the prevention of foodborne infectious disease.

## Results

### A. muciniphila enhances resistance to Listeria monocytogenes infection in mice fed a high-fat diet

**Figure 1.**
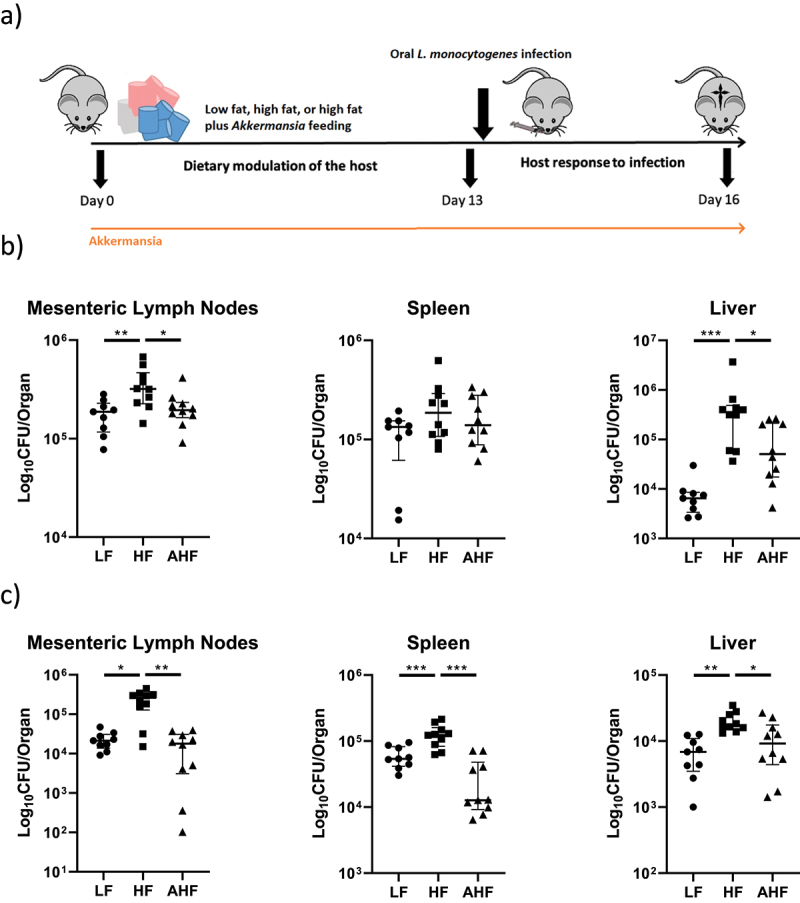
High-fat diet increased susceptibility to oral and systemic *Listeria monocytogenes* infection, but this increase was absent in mice treated with *A. muciniphila*.

Notes: Study timeline (a). Mice were fed a low-fat (LF) or high-fat (HF) diet throughout, while one group was fed a high-fat diet along with gavage with *A. muciniphila* (AHF, *n* = 30 mice per group). Ten mice from each group were sacrificed at day 13 to examine the physiological environment pre-infection, while remaining mice were infected with *Listeria* either by intragastric gavage (IG) or intraperitoneal (IP) injection before sacrifice on day 16. Listerial colony forming units (CFUs) in mice infected by IG were increased by high-fat diet in the mesenteric lymph nodes and liver, and this increase was absent in mice fed a high-fat diet alongside *A. muciniphila* gavage (b, ANOVA and KW-test followed by Tukey’s or FDR pairwise comparisons respectively). In mice infected by IP injection, listerial CFUs were increased by high-fat diet in the mesenteric lymph nodes, spleen, and liver, and this increase was also absent in mice fed a high-fat diet alongside *A. muciniphila* gavage (c, ANOVA and KW-test followed by Tukey’s or FDR pairwise comparisons respectively).

A previous study from our group identified a higher susceptibility to *Listeria* infection in mice fed a HF-diet.^3^ The current study was designed to permit analysis of events occurring in mice directly before *Listeria* infection (day 13 sampling) and during *Listeria* infection (day 16 sampling) (Figure 1a).

Similar to our previous study,^3^ a HF diet significantly increased infectious load in the liver and mesenteric lymph nodes compared to LF mice following intragastric (IG) gavage with the pathogen. However, this increase was not observed in mice fed a HF diet plus *A. muciniphila* gavage (the *Akkermansia* HF (AHF) group) (Figure 1b). When we investigated the effect of diet and *A. muciniphila* treatment on systemic *Listeria* infection, the bacterial load was significantly increased in the spleen, liver, and mesenteric lymph nodes of HF mice compared to LF mice, and this increase was again absent in the AHF group (Figure 1c). Overall, the data demonstrate that *A. muciniphila* functionally improves resistance to *L. monocytogenes* infection in mice fed a HF diet.

### A. muciniphila reduces ileal inflammation and goblet cell production but elevates expression of hepatic immune genes in mice fed a high fat diet

**Figure 2.**
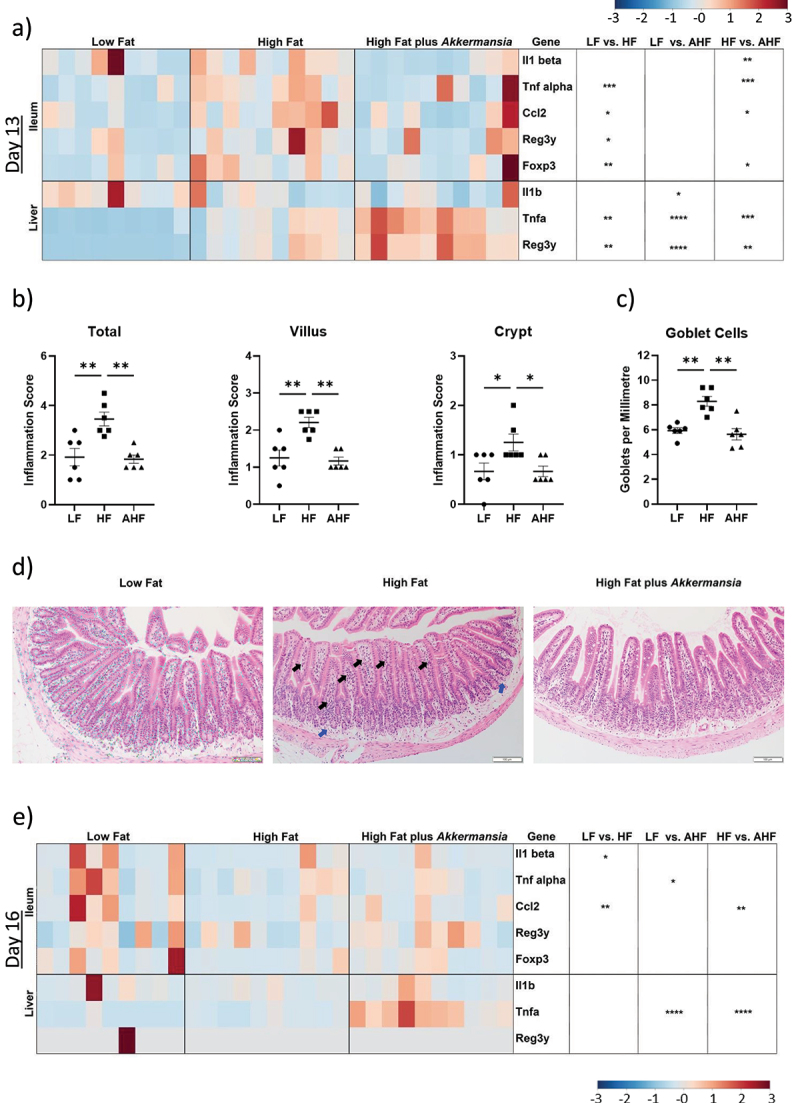
*A. muciniphila* reduces inflammatory expression and goblet cell numbers in the ileum while exacerbating hepatic immune expression.

Notes: Expression of inflammatory genes was measured by qPCR in the distal ileum and liver. This revealed a suppression of inflammatory expression in the ileum, but exacerbation of inflammation in the liver, by *A. muciniphila* at day 13 (a, ANOVA and KW-test followed by Tukey’s or FDR pairwise comparisons respectively, each solid-color rectangle represents one mouse). This was confirmed in the ileum by histological analysis (b, KW-test followed by FDR pairwise comparisons), which also revealed an increase in the number of ileal goblet cells induced by high-fat diet, which was again absent in mice treated with *A. muciniphila* (c, KW-test followed by FDR pairwise comparisons). Representative images of the H&E-stained ileum are presented (d). The arrows highlight the presence of polymorphonuclear cells in the top of the villi (black arrows) and in the bottom of the crypt (blue arrows). qPCR analysis at day 16 revealed a suppression of inflammatory signaling in the context of a high fat diet, while *Ccl2* expression was increased by *A. muciniphila*, which also significantly increased *Tnfα* expression in the liver (e, ANOVA and KW-test followed by Tukey’s or FDR pairwise comparisons respectively, each solid-color rectangle represents one mouse).

To investigate possible mechanisms by which *A. muciniphila* may enhance resistance to *L. monocytogenes* infection in our model, we focused upon changes apparent on day 13, the period directly prior to infection. Changes in host gene expression due to diet, with and without *A. muciniphila*, were measured by qPCR in the distal ileum and liver (Figure 2a). Expression of immune genes *Tnfα, Foxp3* and *Ccl2* was significantly increased in the ileum of mice fed a HF diet compared to LF, consistent with previous studies,^3^ and this increase was absent in HF mice administered *A. muciniphila* (Figure 2a). In addition, *Il1β* expression was increased in the ileum of HF mice compared to AHF, while *Reg3γ* expression was increased in HF relative to LF (Figure 2a).

Histological analysis using H&E-stained ileal sections revealed that ileal inflammation, characterized by poly- and mononuclear cells, was increased by HF diet (as previously demonstrated^3^). However, this infiltration was not observed in mice treated with *A. muciniphila* (Figure 2b,d). This suggests that *A. muciniphila* intervention can moderate inflammation in the small intestine of mice fed a short-term HF diet and that this is coincident with improved resistance to infection. Histological analysis also revealed an increase in goblet cell number in the HF diet group, which was again absent in the AHF group (Figure 2c).

In contrast to the findings in the gut, *A. muciniphila* enhanced diet-induced expression of *Tnfα* and *Reg3γ* genes in the liver of mice fed a HF diet (Figure 2a). *Il1β* expression was significantly lower in AHF mice compared to LF, while both *Tnfα* and *Reg3γ* were increased in HF relative to LF, and AHF relative to HF (Figure 2a). This indicates a suppression of high fat diet-induced expression in the ileum but exacerbation in the liver.

### High fat diet suppresses ileal immune signaling in the context of L. monocytogenes infection, while A. muciniphila increases expression of Ccl2

A suppression of immune signaling in the context of *L. monocytogenes* infection of mice fed a HF diet has been previously reported and may provide a mechanism for the increased susceptibility to infection in these mice.^3^ Here, we similarly observed reduced expression of *Il1β* and *Ccl2* in the ileum of the HF group compared to LF following *Listeria* infection. Interestingly *A. muciniphila* exposure increased expression of *Ccl2* in the HF group to a level similar to LF (Figure 2e).

#### A. muciniphila does not promote significant alterations to the gut microbiota during short-term HF diet feeding

**Figure 3.**
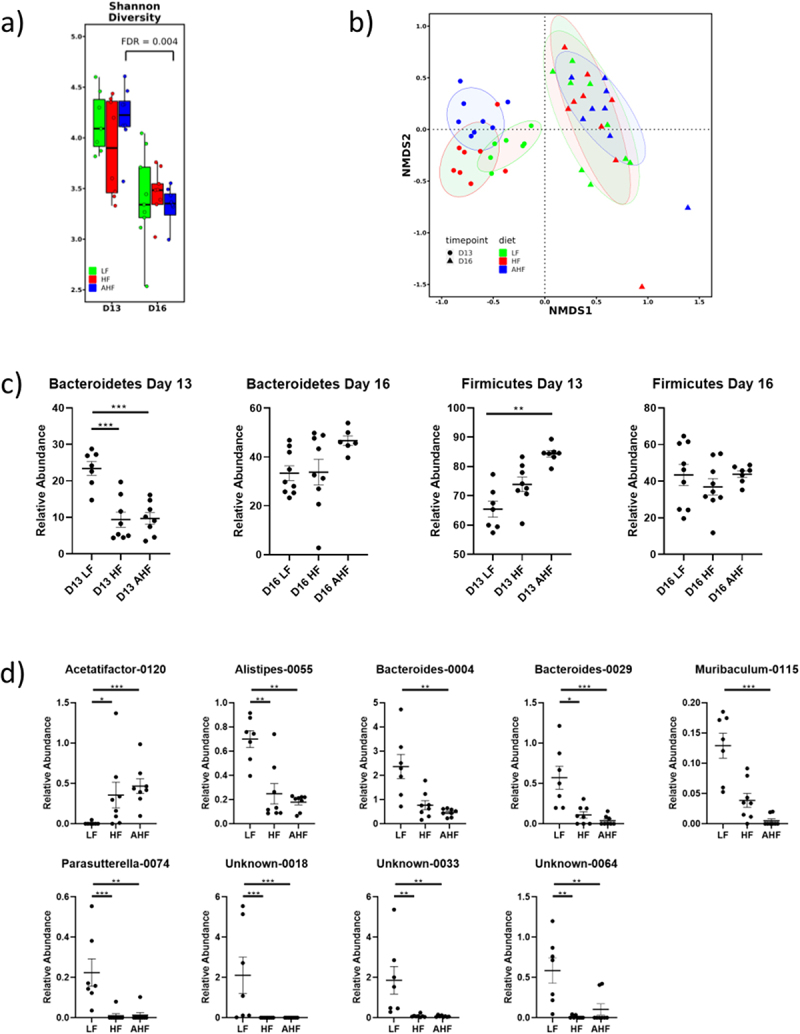
The protective effect of *A. muciniphila* does not appear to be driven by changes in the gut microbiota.

Notes: The gut microbiome was analyzed by 16S gene sequencing. While the alpha diversity decreased over time, there were no changes between groups at any one timepoint (a, ANOVA and KW-test followed by Tukey’s or FDR pairwise comparisons respectively). The beta diversities of all groups were significantly different from each other at day 13, but this difference had disappeared at day 16 (b, PERMANOVA of Bray-Curtis dissimilarity). Relative abundance of the Bacteroidetes phylum was decreased in both high fat and high fat plus *A. muciniphila* (AHF) groups at day 13 compared to low fat (LF), while Firmicutes was increased in AHF compared to LF at day 13, but these differences disappeared at day 16 (c, ANOVA and KW-test followed by Tukey’s or FDR pairwise comparisons respectively). Several taxa were significantly different between groups at day 13 (d, ANOVA and KW-test followed by Tukey’s or FDR pairwise comparisons respectively) and these changes appeared to be driven by diet rather than *A. muciniphila*, while there were no significantly different taxa at day 16.

The gut microbiota provides a barrier between luminal pathogens and the intestinal epithelium and also modulates the intestinal immune response; effects which mediate colonization resistance against incoming pathogens.^21^ We investigated whether the protective effects of *A. muciniphila* against *L. monocytogenes* infection may be mediated through broader effects upon microbiota community structure. There was no significant difference in alpha diversity between groups at day 13 (Figure 3a), while beta diversity of each diet was significantly different from each other diet at day 13 (Figure 3b).

Animals fed a HF diet, with or without *A. muciniphila*, had decreased abundance of Bacteroidetes at day 13, while Firmicutes were increased in AHF mice relative to LF (Figure 3c). These changes are often associated with HF diets, low-grade inflammation, and a reduction in barrier function.^22^ Our data demonstrated that administration of *A. muciniphila* did not significantly alter Bacteroidetes or Firmicutes levels in mice fed a HF diet.

Next, we compared the most abundant 200 taxa for each diet at day 13 and identified significant differences in the abundances of *Alistipes*, *Acetatifactor*, *Bacteroides*, *Muribaculum*, *Parasutteralla*, and several unidentified taxa (Figure 3d). For most taxa, the abundance in LF mice was significantly different to both HF and AHF with no difference between the high fat diets, suggesting that the changes were driven by diet rather than *Akkermansia* administration. In the cases of *Bacteroides-0004* and *Muribaculum-0115*, abundance in AHF alone is significantly lower than in LF. However no significant difference exists between HF and AHF, suggesting that *Akkermansia* does not impact resistance to *Listeria* through overall changes in the composition of the microbiota.

Analysis of the microbiome suggested that although infection with *L. monocytogenes* decreased total bacterial diversity.^13^, there were no significant differences in alpha diversity between treatments on day 16 (Figure 3a), and beta diversity shows no separation between groups (Figure 3b).

The abundances of Bacteroidetes and Firmicutes showed no differences between treatments during infection (Figure 3c). HF diet is usually associated with increased Firmicutes and decreased Bacteroidetes phyla, as we saw at day 13, which suggests that the concordance of phyla may reflect a homogenizing effect of *Listeria* infection. Similarly, at genus level, there were no significant differences at day 16 after correction for multiple comparisons (data not shown, ANOVA and KW-test followed by Tukey’s or FDR pairwise comparisons respectively, *p* > 0.05). Overall, we determined a minimal effect of *A. muciniphila* upon the microbiota community prior to infection with the treatment group (AHF) resembling the group fed HF diet alone. During infection we demonstrated a concordance of all mouse groups reflecting a potentially dominant impact of infection on microbiota community structure.

### Changes in fecal and cecal metabolites are primarily driven by diet

**Figure 4.**
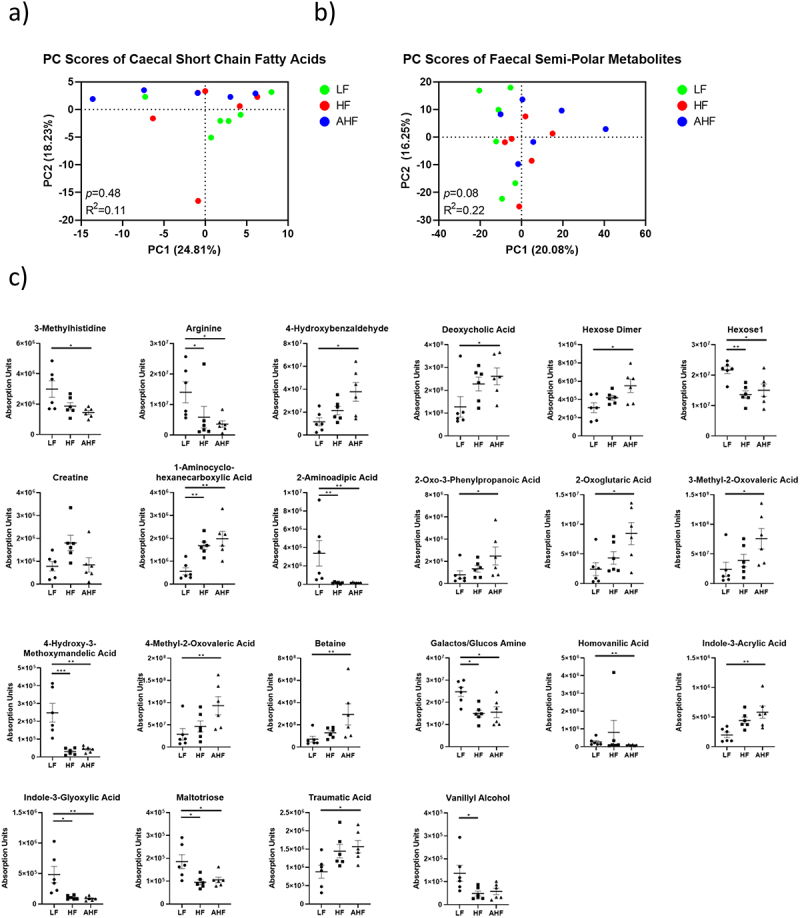
The protective effect of *A. muciniphila* does not appear to be driven by fecal or cecal metabolomes at day 13.

Notes: The production of microbial metabolites was examined by measuring short chain fatty acids in the cecum and semi-polar metabolites in the feces of each group by UPLC at day 13. Principal component analysis detected no changes between groups for cecal or fecal metabolites (a and b, Principal Component Analysis). Only the significantly different metabolites, identified at least to a level based on accurate mass and known retention time obtained from standards analyzed on the same system, are presented (c, ANOVA and KW-test followed by Tukey’s or FDR pairwise comparisons respectively).

To investigate the changes of microbial metabolites provoked by HF diet and *A. muciniphila* treatment before and after *Listeria* infection, we performed targeted analysis of short chain fatty acids in the cecal contents, and untargeted analysis of semi-polar metabolites in the feces, from six randomly selected mice per group on day 13. Principal component analysis (PCA) found no differences between the groups in the cecum or feces (Figure 4a,b). There were no significant differences detected among SCFAs in the cecum (Supplemental Figure 1). In the feces, there were no changes detected between HF and AHF mice (Figure 4c), suggesting that the protective effect of *A. muciniphila* administration is not mediated through these metabolites.

## Discussion

A Westernized diet comprising a high caloric intake from fats and low consumption of fermentable fiber has been associated with diseases such as inflammatory bowel disease, obesity and type 2 diabetes.^1^ A Westernized diet can also compromise intestinal barrier function and broadly influence immune signaling. Indeed, recent work by our group demonstrated an increased susceptibility to gastrointestinal and systemic infection by *L. monocytogenes* in mice fed a high fat (HF) diet over a relatively short (2 week) time period.^1,3^ We demonstrated significant alterations to intestinal histology and inflammatory status by short-term administration of a HF diet which were co-incident with predictable alterations to the microbiota (increased Firmicutes and decreased Verrucomicrobia).^3^ However, it was unclear whether the effects on host immunity were mediated by diet or microbiota, or whether particular components of the microbiota could provide protection against infection in the appropriate context. Given that *A. muciniphila* is negatively impacted by HF diet and has been associated with positive effects on host barrier function, we examined the role of *A. muciniphila* upon host susceptibility to infection in the context of a HF diet.

*A. muciniphila* has been consistently associated with positive health benefits in models of metabolic disease, diabetes, and obesity.^23^ Levels of *A. muciniphila* in the gut correlate negatively with bodyweight in humans, while *Akkermansia* supplementation in mice reversed HF diet-induced weight gain, hyperlipidemia, inflammation, and intestinal permeability.^18,23^ In our short-term (pre-obesity) model, HF diet increased expression of inflammatory markers but inflammation was reduced significantly by gavage with *A. muciniphila*. Previous studies have demonstrated the broadly anti-inflammatory effects of *A. muciniphila* intervention in long-term dietary models that reflect development of obesity or type 2 diabetes.^23,24^ Our current data suggest that such effects are demonstrable even after a shorter time period (pre-obesity) and correlate with an improvement in resistance to *L. monocytogenes* infection.

In contrast to the suppression of the inflammatory markers observed in the ileum, *A. muciniphila* enhanced inflammatory expression in the liver. Levels of *Tnfα* and *Reg3γ* expression were higher in the livers of HF mice compared to LF and were increased further in AHF mice. This was an unexpected finding which may reflect the short-term nature of our study, as *A. muciniphila* has been reported to decrease TNFα in a mouse model of liver injury,^25,26^ and reduced inflammation in a model of *C. difficile* infection.^27,28^ In our infectious model it is possible that the elevated expression of *Tnfα* in the liver can enhance systemic resistance to the pathogen. Indeed, it is interesting to note that these high levels of expression were maintained during *Listeria* infection (day 16) when a reduced *Listeria* load was observed (Figure 1c). Generally, the host response in the ileum during infection (day 16) was similar between mice fed a HF diet and those that received a HF diet plus *A. muciniphila*. This involved a suppression of immune signaling in mice fed a high fat diet during *Listeria* infection which has been previously reported and may contribute to the susceptibility to infection.^3^ One exception to this was an increase in expression of *Ccl2* in mice fed a HF diet plus *Akkermansia* compared to the HF group. CCL2-mediated stimulation of monocytes’ CCR2 chemokine receptor plays a vital role in the innate immune response to *Listeria* infection,^29^ suggesting that modulation of innate immunity could contribute to *Akkermansia*-induced resistance to this pathogen.

To determine whether *A. muciniphila* administration affected microbiota community structure in our model we profiled the gut microbiota using 16S amplicon sequencing both before and during *Listeria* infection. Analysis demonstrated that HF diet increased the Firmicutes phylum and reduced the Bacteroidetes as noted in our previous short-term feeding study^3^ and in numerous longer term murine obesity studies.^30,31^ However, in our model *A. muciniphila* administration did not alter the Firmicutes or Bacteroidetes profiles over a two-week period. Similarly, whilst studies following longer-term administration of *A. muciniphila* have reported effects on specific bacterial interactions in the gut microbiota,^32^ we failed to determine significant impact of *A. muciniphila* upon individual taxa. We suggest that greater impact of *A. muciniphila* upon the microbiota community structure in the context of a HF diet may be time-dependent. Surprisingly, there were no significant differences in the endogenous abundances of *Akkermansia* detected between LF and HF groups, yet infectious load was still increased by high fat diet. This suggests that the mechanisms by which HF diet increases susceptibility to *Listeria* infection involve more than the suppression of *A. muciniphila*, despite the protective effect provided by gavage with this species outlined in the current study.

In addition, extensive profiling of bacterial metabolites demonstrated that *A. muciniphila* administration did not significantly alter the metabolome relative to mice fed a HF diet alone. Overall, the findings suggest that the effects of *A. muciniphila* upon host immunity and anti-*Listeria* functions are independent of broader changes to microbiota community structure in the gut and are likely to be a direct consequence of the interaction between *A. muciniphila* and the host GI tract. Several studies have examined the molecular effectors of this interaction which is mediated through the *A. muciniphila* proteins AMUC_1100 and P9.^2,33^ Indeed, studies have demonstrated that pasteurized *A. muciniphila* or purified AMUC_1100 can exert similar effects on the host when compared to live bacterial cultures.^33,34^ Future work in our lab will examine the potential for pasteurized *A. muciniphila* or a protein extract to exert an anti-infective effect.

To our knowledge this represents the first report of the effects of *A. muciniphila* administration upon the outcome of a foodborne infectious pathogen. Our findings suggest a specific interaction between *A. muciniphila* and the host gut that modulates local inflammatory profiles and the gastrointestinal environment but does not depend upon alterations to the broader microbial community structure. The findings build upon previous work that has identified particular taxa that contribute to colonization resistance against *L. monocytogenes* infection^35^ toward the development of microbial interventions to prevent or reduce foodborne infection.

## Materials and methods

### Reagents

C57BL/6J mice (Envigo, UK); low fat chow (DIO series diets D12450H, Research Diets, Inc., USA); high fat chow (DIO series diets D12451, Research Diets, Inc., USA), GenElute Mammalian Total RNA kit (Sigma, USA), ReadyScript cDNA Synthesis Mix (Sigma, USA); KiCqStart SYBR Green qPCR ReadyMix (Sigma, USA); QIAamp Fast DNA Stool Kit (Qiagen, Germany); Custom oligo qPCR primers, (Eurofins Genomics, Germany). *A. muciniphila* was grown every day in 10 ml aliquots of anaerobic Mucin v3 media (10% inoculation) at 37°C. Nitrogen (and boiling) was used to remove the presence of oxygen from Mucin v3 media. Mucin v3 media contains peptone (Fluka), yeast extract (Roth), KH2PO4 (Fluka), NaCl (Fluka), (NH4)SO4 (Acros organics), MgSO4 (Acros organics), CaCl2 (Acros), NaHCO3 (Fluka), D-glucose (Fischer chemical), porcine mucin type II (Sigma), porcine hemin (Acros organics), L-cysteine (Sigma), and water.

### Animals and study design

All the animal procedures were carried out in agreement with the guidelines of the European Commission for the handling of laboratory animals (directive 2010/63/EU), under authorizations issued by the Health Products Regulatory Authority (HPRA, Ireland) for the use of animals for scientific purposes and approved by the Animal Experimentation Ethics Committee of University College Cork.

Seven-week-old C57BL/6J mice were randomly assigned to low fat (LF) diet, high fat (HF) diet, or high fat diet plus *A. muciniphila* (AHF) treatments (*n* = 30 per group, Figure 1). Mice were housed in a specific pathogen-free facility on a 12-hour light/dark cycle at 22°C with access to water and either LF or HF chow *ad libitum*. *A. muciniphila* (1×10^9^ CFUs in 200 μL) was delivered by daily intragastric gavage throughout the experiment. At day 13, *n = 10* mice per group were sacrificed, while the remaining mice were treated with *L. monocytogenes* EGD-e InlA^m^ in PBS either by intragastric gavage (5×10^9^ CFUs, *n = 10* per group) or intraperitoneal injection (5×10^5^ CFUs, *n = 10* per group), before sacrifice on day 16. Internal organs were homogenized and CFUs per organ measured on *brain heart infusion* (BHI) agar plates.

### Quantitative real-time PCR

RNA was extracted from the distal ileum using the GenElute Mammalian Total RNA kit (Merck) as per manufacturer instructions and converted to cDNA using the ReadyScript cDNA Synthesis Mix (Sigma). qPCR was performed using KiCqStart SYBR Green qPCR ReadyMix (Sigma) in a LightCycler 480 for 40 PCR cycles using appropriate primers. Relative transcription was calculated using the 2-ΔΔCT method standardized to the average of the low fat group ΔCT. Primer sequences are presented in Supplemental Table 1.

### Histological analysis

Tissue from the distal ileum was fixed in 4% paraformaldehyde overnight and dehydrated with 70% ethanol for 72 h at 4°C prior to paraffin embedding. 5 μm sections were mounted into slides and stained with hematoxylin and eosin using a standard procedure. Slides from six mice per group were blinded and inflammation was scored by an independent researcher (S.M.) using a protocol adapted from Drolia et al.^36^ with some modifications.^3^ Samples were scored on a scale of 0–3 for two parameters: infiltration of inflammatory cells (mostly mononuclear cells) to the villi and infiltration of mono- and polymorphonuclear cells to the crypt, yielding a maximum score of 6. In our model, polymorphonuclear cells were mainly located at the bottom of the crypts. The gradient of the inflammatory cell infiltration was based on 3 = highly increased, 2 = moderately increased, 1 = mildly increased and 0 = normal. Pictures were captured using an Olympus B×53 widefield microscope with an Olympus DP74 digital camera (Olympus GmbH, Germany). For goblet cell enumeration, slides from six mice per group were blinded, the lengths of all intact villi were measured and the number of goblet cells per villus was counted. This gave the number of goblet cells per millimeter. A minimum of 15 villi were measured per mouse, with a mean average of 34 villi included.

### Fecal 16S rRNA gene sequencing

Fecal pellets were collected from each mouse on the day of sacrifice. DNA was extracted using the QIAamp Fast DNA Stool Kit as per the manufacturer’s instructions with the addition of a bead-beating step. The V3-V4 (341F, 805 R) variable region of the 16S rRNA gene was amplified according to the 16S metagenomic sequencing library protocol (Illumina, San Diego, CA, USA) and sequenced on an Illumina MiSeq. Raw sequences were profiled using FastQC/MultiQC^37,38^, adapter sequences removed using cutadapt (minimum length 200bp, maximum error rate of 0.2)^39^, before optimizing filtering parameters in FIGARO^40^, then applying optimized filtering and resolving amplicon sequence variants (ASVs) in R library DADA2 (0 mismatches, minimum overlap of 20bp, individual pools, consensus removal of bimeras).^41^ Taxonomy was assigned to ASVs using DADA2’s assignSpecies function (100% sequence identity) with reference to the SILVA database (release 138^42^), before removal of contaminant sequences using R library decontam.^43^ All downstream analysis was performed in R version 3.4.3.^44^ Alpha and beta diversities were calculated using the R package *phyloseq*. Differences in alpha diversity were assessed using the Mann-Whitney test. Differential abundant analysis was performed using DESeq2.^45^ The *adonis* function in the vegan library was used to assess group-level differences in the microbiota.^46^

### Ultra-Performance Liquid Chromatography – Mass Spectrometry (UPLC-MS)

The fecal and cecal metabolome was measured at day 13 to investigate the production of microbial metabolites. Cecal content was examined for short chain fatty acids (SCFAs) and fecal samples were examined for semipolar metabolites, from six mice randomly selected from each treatment group.

Sample analysis was carried out by MS-Omics as follows. For SCFAs, samples were acidified using hydrochloride acid, and deuterium labeled internal standards where added. All samples were analyzed in a randomized order. Analysis was performed using a high polarity column (Zebron™ ZB-FFAP, GC Cap. Column 30 m x 0.25 mm x 0.25 µm) installed in a GC (7890B, Agilent) coupled with a quadrupole detector (5977B, Agilent). The system was controlled by ChemStation (Agilent). Raw data was converted to netCDF format using Chemstation (Agilent), before the data was imported and processed in Matlab R2014b (Mathworks, Inc.) using the PARADISe software described by Johnsen et. al.^47^ For semi-polar metabolites, the analysis was carried out using a Thermo Scientific Vanquish LC coupled to Thermo Q Exactive HF MS. An electrospray ionization interface was used as ionization source. Analysis was performed in negative and positive ionization mode. The UPLC was performed using a slightly modified version of the protocol described by Catalin et al.^48^ Peak areas were extracted using Compound Discoverer 3.1 (Thermo Scientific). Identification of compounds were performed at four levels; Level 1: identification by retention times (compared against in-house authentic standards), accurate mass (with an accepted deviation of 3ppm), and MS/MS spectra, Level 2a: identification by retention times (compared against in-house authentic standards), accurate mass (with an accepted deviation of 3ppm). Level 2b: identification by accurate mass (with an accepted deviation of 3ppm), and MS/MS spectra, Level 3: identification by accurate mass alone (with an accepted deviation of 3ppm).

### Statistics

Statistics were performed in SPSS Version 28 (Chicago, IL, USA) and GraphPad version 9 (San Diego, CA, USA). Statistical significance was set to *p* < 0.05. Normality was determined by the Shapiro-Wilk test. Groups were compared with one-way ANOVA or Kruskal-Wallis (KW) test as appropriate, followed by Tukey’s or the two-stage step-up method of Benjamini, Krieger and Yekutieli pairwise comparisons respectively. Outliers were identified as values 2.2-times the interquartile range above or below the third or second quartile respectively. Permutational ANOVA (PERMANOVA) was used to compare β-diversity via Bray-Curtis dissimilarity, while principal component analysis was used to investigate UPLC data. Throughout, asterisks denote significance where * represents *p* < 0.05, ** *p* < 0.01, *** *p* < 0.001, and **** *p* < 0.0001.

## Data Availability

All data are available on Zenodo at the following link https://doi.org/10.5281/zenodo.7590374.
